# Cost–Benefit and Cost–Utility Analyses to Demonstrate the Potential Value-for-Money of Supermarket Shelf Tags Promoting Healthier Packaged Products in Australia

**DOI:** 10.3390/nu14091919

**Published:** 2022-05-03

**Authors:** Jaithri Ananthapavan, Gary Sacks, Liliana Orellana, Josephine Marshall, Ella Robinson, Marj Moodie, Miranda Blake, Amy Brown, Rob Carter, Adrian J. Cameron

**Affiliations:** 1 Deakin Health Economics, School of Health and Social Development, Institute for Health Transformation, Deakin University, Geelong 3220, Australia; marj.moodie@deakin.edu.au (M.M.); rob.carter@deakin.edu.au (R.C.); 2 Global Obesity Centre, School of Health and Social Development, Institute for Health Transformation, Deakin University, Geelong 3220, Australia; gary.sacks@deakin.edu.au (G.S.); josephine.marshall@deakin.edu.au (J.M.); ella.robinson@deakin.edu.au (E.R.); miranda.blake@deakin.edu.au (M.B.); adrian.cameron@deakin.edu.au (A.J.C.); 3 Biostatistics Unit, Faculty of Health, Deakin University, Geelong 3220, Australia; l.orellana@deakin.edu.au; 4 City of Greater Bendigo Council, Bendigo 3550, Australia; amybrownprofessional@gmail.com

**Keywords:** cost–benefit analysis, cost–utility analysis, supermarket, healthy retail, shelf tag, economic evaluation

## Abstract

The supermarket environment impacts the healthiness of food purchased and consumed. Shelf tags that alert customers to healthier packaged products can improve the healthiness of overall purchases. This study assessed the potential value-for-money of implementing a three-year shelf tag intervention across all major supermarket chains in Australia. Cost–benefit analyses (CBA) and cost–utility analyses (CUA) were conducted based on results of a 12-week non-randomised controlled trial of a shelf tag intervention in seven Australian supermarkets. The change in energy density of all packaged foods purchased during the trial was used to estimate population-level changes in mean daily energy intake. A multi-state, multiple-cohort Markov model estimated the subsequent obesity-related health and healthcare cost outcomes over the lifetime of the 2019 Australian population. The CBA and CUA took societal and healthcare sector perspectives, respectively. The intervention was estimated to produce a mean reduction in population body weight of 1.09 kg. The net present value of the intervention was approximately AUD 17 billion (B). Over 98% of the intervention costs were borne by supermarkets. CUA findings were consistent with the CBA—the intervention was dominant, producing both health benefits and cost-savings. Shelf tags are likely to offer excellent value-for-money from societal and healthcare sector perspectives.

## 1. Introduction

In Australia, 67% of adults and 25% of children and adolescents were living with overweight or obesity in 2017–2018 [1]. Obesogenic food environments driving poor diet quality have been identified as a key factor contributing to the high prevalence of elevated body mass index (BMI) and its associated negative health outcomes [2,3]. Food retail environments influence customer choices [4], and with 63% of all food spending in Australia being in supermarkets [5], they have great potential to impact population diets.

Evidence of the effectiveness of supermarket-based interventions to improve the healthiness of food purchases has increased over the last 10 years [6,7]. Recent systematic reviews have found that the majority of interventions resulted in positive changes to the healthiness of food purchases [6,7]. Shelf labelling systems (referred to as ‘shelf tags’ hereafter) in supermarkets are used to highlight to customers the nutritional content of packaged foods. Previous research has demonstrated that adding easy-to-interpret nutrient summary scores to supermarket price labels on shelves has been effective in increasing the healthiness of products purchased in both experimental and real-world trials [8,9,10,11,12,13,14], although a recent European study reported mixed findings [15]. No previous studies have reported the effect on purchasing of larger, promotional shelf tags that highlight only the healthiest packaged products.

The Australian and New Zealand Health Star Rating (HSR) system is a voluntary front-of-pack labelling scheme, endorsed by the Australian and New Zealand governments, that assigns health stars between 0.5 (least healthy) and 5 stars (most healthy) based on the nutrition profile of packaged products [16]. Since the HSR scheme was endorsed in 2014, there has been limited but increasing levels of implementation, with approximately 14% of eligible products displaying the HSR on front-of-pack in 2015, rising to 41% in 2019 [17,18]. We conducted a 12-week non-randomised controlled trial that tested the impact on sales of installing promotional shelf tags for all products scoring 4.5 or 5 stars according to the HSR system (referred to as the ‘shelf tag study’ hereafter). The shelf tag study was undertaken in 2015 across three intervention and four control supermarkets within one supermarket chain in regional Victoria, Australia. The study found a significant increase in the proportion of healthier packaged foods sold with HSR of 4.5 or 5 in intervention stores compared to control stores [19].

Evidence for the effectiveness of shelf tag interventions to improve the healthiness of food purchases in supermarkets is increasing. However, there is currently no evidence of their cost-effectiveness to inform decision-makers of whether shelf tag interventions that are potentially effective from a public health perspective also offer good value-for-money [20]. Cost–utility analysis (CUA), where the outcome of an intervention is valued using a generic preference-based health measure that captures the quantity and quality of life improvements, is the most commonly used economic evaluation technique in the health sector and is required for health technology assessments in Australia [21,22]. However, preventive health interventions in settings such as supermarkets have impacts on non-healthcare stakeholders and therefore the value of a healthcare sector perspective is limited, and there is growing recognition that CUA does not adequately capture the credentials of these interventions [23,24]. Cost–benefit analysis (CBA) is an economic evaluation technique where the costs and benefits of an intervention are monetised. Cost–benefit analysis is more conducive to the adoption of a broader perspective and aims to assess whether the costs of an intervention, borne by all members of society, are outweighed by the societal benefits. For interventions with impacts outside the healthcare sector, CBA may have advantages over CUA where benefits are generally limited to health impacts [25]. It has been suggested that CBA may be a better technique to evaluate nutrition interventions, such as nutrition labelling, which are likely to have cost implications on the private sector [26]. Despite this, CBA is rarely undertaken for health-related interventions [27]. Presenting both CBA and CUA analyses may allow decision-makers to better understand the similarities and differences between them and how they might be applied to health-related resource allocation decisions [28].

The aim of this study was to use the evidence of costs and effectiveness that was collected as part of an Australian shelf tag study to assess the economic credentials of implementing the shelf tag intervention across the four largest supermarket chains in Australia. Both CBA and CUA were undertaken, using societal and healthcare sector perspectives respectively. The CBA and CUA consistently found that the intervention was likely to be cost-effective. The costs were predominantly borne by the supermarket industry whereas the main beneficiaries of the intervention were individuals and the Australian federal government.

## 2. Materials and Methods

### 2.1. The Intervention

Details of the shelf tag study and results have been reported separately [19]; with the key features outlined here. The shelf tag intervention was designed in collaboration with public health researchers, a supermarket retailer (Champions IGA) and a local government (City of Greater Bendigo). The principles used for intervention development included low cost, feasibility of implementation, scalability, not likely to adversely affect retailer profits, and likely impact on population diets [29]. All seven trial supermarkets (three intervention stores and four control stores) were part of the Champions IGA supermarket group and all were located in regional Victoria, Australia. The study was undertaken with a four-week baseline period followed by an eight-week intervention period. During the intervention period, intervention stores displayed a colourful shelf tag (7 × 10 cm, see Supplementary Materials, Figure S1) directly in front of products that were classified as having an HSR of 4.5 or 5. The shelf tags were displayed for all eligible products regardless of whether they displayed an HSR on the product packaging.

Weekly sales data (number of units and costs) were collected from the intervention and control stores. The primary outcome measure of the trial was percentage change in the units and weight of eligible products (those with HSR of 4.5 or 5) sold relative to all packaged foods sold and was calculated using difference-in-difference analyses [19]. The sales data were matched to product nutrient composition using the 2015 Australian FoodSwitch^®^ [30] and the AUSNUT 2011–2013 food nutrient [31] databases. This enabled a difference-in-difference analysis of the change in the energy density (kJ/100 g) of packaged foods sold during the intervention period. The results showed that there was a reduction in the energy density of packaged foods sold in the intervention stores compared to control stores of 67 kJ/100 g (Kruskal–Wallis test *p* = 0.034), representing a 13.3% (SE: 5.2%) reduction in the energy density of packaged foods sold.

The economic evaluation estimated the potential costs and benefits of the shelf tag intervention if implemented as a voluntary initiative by state and territory governments in Australia as part of their obesity prevention strategies. Given the limited evidence on the feasibility of maintaining this intervention over the longer term, it was assumed that the intervention was implemented for three years. It was assumed that the intervention targeted the four leading supermarket chains in Australia (Woolworths, Coles, Aldi and Metcash Limited (umbrella company for independently owned IGA stores)) representing 84% of market share [32]. Given the limited data on whether supermarket chains will take up the voluntary initiative, various scenarios of intervention uptake were tested. The analyses were based on the data from the shelf tag study and adjusted to represent the scale up of the intervention across other supermarket retailers.

### 2.2. Modelling the Intervention Impact on Population Energy Intake and Weight

**Figure 1:**
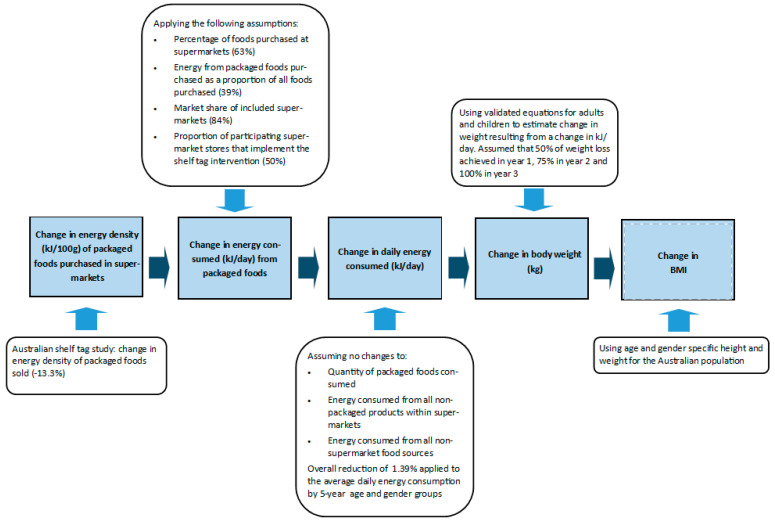
Logic pathway of intervention effect on average population weight and body mass index. Notes: BMI: body mass index; g: grams; kg: kilogram; kJ: kilojoules.

The program logic of how the shelf tag intervention was modelled to impact population body weight and body mass index (BMI) is shown in Figure 1. It was assumed that the 13.3% reduction in the energy density of packaged foods sold would translate to the same percentage reduction in the energy consumed from packaged foods. For this assumption to hold, it was assumed that the quantity of packaged foods consumed (in kg) stayed the same and the energy consumed from all other products within and external to the supermarket remained unchanged with the intervention. The proportion of dietary intake from packaged foods as a proportion of all foods purchased in supermarkets was estimated from the baseline sales data from the shelf tag study (39%). This was done by calculating the energy (kJ) purchased from packaged food products eligible for the HSR as a proportion of the total energy of all food products purchased (both eligible and not eligible for HSR). The effect size was adjusted to account for the proportion of foods purchased at supermarkets (63%) and the market share (84%) of participating supermarkets [5,32]. In the primary analyses, it was assumed that the intervention would be implemented in the four targeted supermarket chains. This assumption was based on the evidence that these supermarket chains are leading the voluntary uptake of the front-of-pack HSR scheme on their private label products, indicating that there is appetite for these type of interventions amongst the largest supermarket retailers in Australia [18] (See Supplementary Materials, Table S1). We selected an implementation rate of 50% in each supermarket chain, based on an assumption that voluntary implementation may not result in high levels of compliance across all stores due to variations in store format and geographic location. After all assumptions were applied, we calculated that the intervention would result in a daily energy intake reduction of 1.39%. 

The percentage change in energy intake was applied to average daily energy intake by five-year age and gender groups estimated from the 2011–2012 Australian Health Survey [33] (the latest nationally-representative survey with 24 hour recall of dietary intake). The estimated change in average daily energy intake was translated to a change in weight using validated equations for adults [34] and children [35]. According to the studies underpinning these equations, it is predicted that with a persistent daily energy deficit, 50% of the total predicted weight loss occurs at 1 year, 75% at 2 years, and 100% at three years [34]. The change in weight was quantified as changes in BMI using five-year age and gender-specific height and weight for the Australian population in 2017–2018 [36,37]. The estimated change in average daily energy intake was assumed to persist for the duration of the three-year intervention (Figure 1).

### 2.3. Modelling the Cost of the Intervention

#### 2.3.1. Supermarket Costs

The cost to supermarkets included shelf tag design, matching the nutrient profile of packaged foods sold in each supermarket to identify the products eligible for the shelf tag, printing, installation, monitoring, and replacement (Supplementary Materials, Table S1). The number of products in each of the supermarket chains was taken from a 2018 industry report [32]. This was adjusted to include the expected number of new products that would be added in years two and three of the intervention. Given that each IGA store is independently owned, product identification codes vary across stores; therefore, it was assumed that 70% of products would need to be matched to the HSR scheme at each store. For the other supermarket companies that have a centralised system to manage product lists, codes and sales data, the matching process was assumed to be done at a company level (with no additional costs for each additional store for product matching). Cost data were based on the shelf tag study and adjusted to other supermarket chains based on the number of products sold by each supermarket chain, the average floor space per store for the shelf tag stores relative to the other supermarket chains, and the number of stores in each chain [32,38]. We assumed that the cost of the intervention to supermarkets was not passed onto customers through higher prices.

#### 2.3.2. Government Costs

It was assumed that each state and territory government would encourage this voluntary supermarket initiative by providing guidance and support to the supermarket industry regarding implementation. We assumed this would involve meetings with the company head office in each state to discuss the initiative, provide advice on product matching to HSR and to provide support at the store level (involving store visits in year 1). Resource use was based on staff time accrued by the NSW Government in supporting fast food companies to implement menu labelling changes [39] (Supplementary Materials, Table S1).

Personnel time costs were valued using average wages for various roles reported by the Australian Bureau of Statistics and included salary on-costs (13%) [40] and 17.5% leave loading [41]. All costs were adjusted to AUD 2019 values using various official indices (Total Health Price Index [42], Wage Price Index [43], CPI for food and non-alcoholic beverages [44] and Gross Domestic Product Index [42], see Supplementary Materials, Table S1).

### 2.4. Overview of Cost-Effectiveness Modelling

The specifications for the CBA and the CUA are shown in Table 1. The CBA was undertaken from a societal perspective using specifications based on a published CBA framework for preventive health interventions [45]. The CUA was undertaken from a healthcare system perspective using specifications as outlined by the Australian Pharmaceutical Benefits Advisory Committee (PBAC) [21] for health technology assessments.

The supermarket shelf tag intervention was compared to a ‘status quo’ comparator (the comparator is often referred to as the base case in CBA [45]). The comparator population was identical to the intervention population except for the changes in BMI profile resulting from the intervention. The time horizon for capturing the health gains and healthcare cost impacts of the three-year intervention was the lifetime of the population (or 100 years). A lifetime time horizon is appropriate as even short-term changes in population dietary intake can influence the epidemiology of obesity related diseases over the longer-term at a population level.

The ACE-Obesity Policy model, a proportional, multi-state life table Markov cohort model, was used to estimate the impact of the change in BMI resulting from the shelf tag intervention on long-term health outcomes (measured in health-adjusted life years (HALYs)) and healthcare costs. Details of the model have been previously published [46,47] and are described here briefly. The MS Excel-based model was recently updated and simulates the 2019 Australian population (aged 2–100 years) [48]. It estimates the impact of changes in population weight on the incidence of nine diseases causally related to BMI (kidney, endometrial, breast, and colorectal cancer, type 2 diabetes, stroke, hypertensive heart disease, ischaemic heart disease and osteoarthritis of the hip and knee) using relative risks from the Global Burden of Disease study [49]. Individual disease models with four health states (‘healthy’, ‘diseased’, ‘dead from the disease’, and ‘dead due to other causes’) predict the impact of the change in disease incidence on prevalence and mortality. The disease models were populated with disability weights from the 2017 Global Burden of Disease study to capture the morbidity impact of the intervention [50]. Population-level changes in the morbidity and mortality associated with the nine diseases were aggregated as HALYs. Costs associated with each of the diseases were provided by the Australian Institute for Health and Welfare for previous studies [51,52]. Given the lack of more contemporary published data, the 2001 and 2016 costs were inflated to 2019 values using the Total Health Price Index [42,53]. 

All costs and benefits are presented in 2019 values. The Consolidated Health Economic Evaluation Reporting Standards checklist [54] is provided in Supplementary Materials, Table S2.

#### 2.4.1. Cost–Benefit Analysis Modelling

CBA requires health and non-health benefits to be valued in monetary terms [28]. The HALY gain was monetised using a recent estimate of the value of a statistical life year (VSLY) recommended for use in government policy-making in Australia [55].

The installation of shelf tags offers additional nutrition information in order for customers to meet their own preferences. This is considered a consumer benefit in addition to the nutrition benefits resulting from customers choosing products with higher HSR values [56,57]. A recent Australian study by Cooper et al. [58] reported that 48% of shoppers reported either agreeing or strongly agreeing with the statement: ‘A Health Star Rating would make it easier for me to make selections when shopping’. Cooper and colleagues [58] aimed to estimate consumer willingness to pay for packaged foods with the HSR system using a double-bounded dichotomous choice contingent valuation model. On average, participants were willing to pay a price premium of AUD 0.11 or 3.67% of the price of the initial product, for the same product with the HSR, representing consumer surplus [58]. Although data on the average annual household spend on groceries are available (AUD 6974 [59]), the proportion of total grocery spend on packaged foods is unknown. We assumed that the proportion of energy from packaged foods purchased as a proportion of all foods purchased (from the shelf tag study, 39%) was indicative of the proportion of annual household grocery spend on packaged foods (calculated to be approximately AUD 2752). This was further adjusted to reflect the proportion of packaged foods eligible for 4.5 and 5 HSR (from the shelf tag study and other supermarket audits [17]) and the proportion of households impacted by the intervention (see Supplementary Materials, Table S1). All costs and benefits were discounted uniformly using a 3% discount rate for the primary analysis as recommended by the CBA framework for preventive health interventions [45].

Systematic reviews of the impact of healthy food retail interventions on retailers [60,61,62], customer exit surveys from the shelf tag study [19], and a process evaluation undertaken by Blake et al. [63] of a larger healthy supermarket trial in Australia, informed the assessment of the impacts on retailers. The study by Blake et al. [63] was conducted in the same supermarket chain (IGA) and included shelf tags as part of a multi-component healthy supermarket intervention. The main impacts on retailers relate to (i) customer perceptions including customer satisfaction with the intervention and store environment, and demand for the promoted products; (ii) commercial viability, which included a diverse range of profitability outcomes; and (iii) retailer perceptions, which included staff satisfaction and perceptions on ease of intervention implementation. Measurement issues limited the monetary valuation of these impacts on retailers; therefore, they are not incorporated into the outcomes of the CBA, but the quantitative and qualitative assessment of these impacts should be considered alongside the CBA results.

As recommended by the CBA framework for preventive health interventions [45], the net present value (NPV = net present value of benefits − net present value of costs) and the benefit–cost ratio (BCR = net present value of benefits/net present value of costs, see Supplementary Materials, Table S3 for equations) were calculated. All outcomes or impacts of the intervention, including the healthcare cost-savings, were accounted for on the benefits side of the equation [45]. Disaggregated costs and benefits by stakeholder group are presented in a balance sheet format to identify trade-offs between groups [28].

#### 2.4.2. Cost–Utility Analysis Modelling

The CUA results are presented on a cost-effectiveness plane. The incremental cost–effectiveness ratio (ICER) was calculated by dividing the net present value of costs of the intervention by the net present value of HALYs gained. Based on previous funding decisions by the Australian PBAC [64], and aligned with other Australian economic evaluations of obesity prevention interventions [47], the cost-effectiveness threshold was assumed to be AUD 50,000 per HALY gained. All costs and benefits were uniformly discounted at 5% [65] as recommended by PBAC guidelines [21].

#### 2.4.3. Sensitivity Analyses

The impact of alternative parameter values and assumptions on the cost-effectiveness results were tested in one-way and multi-way sensitivity analyses (Table 2, further details are provided in Supplementary Materials, Table S4). The key variables/assumptions tested in both the CBA and CUA included the discount rate, length of the intervention, duration of intervention effect, uptake by supermarket chains, and the impact of mandatory implementation (through government regulation) rather than a voluntary initiative. For the CBA, additional sensitivity analyses assessed the removal of the consumer surplus benefit, shorter time horizon and various estimates for valuing the health benefits of the intervention. For the CUA, an additional analysis was undertaken using the reference case specifications outlined by the Second Panel on Cost-Effectiveness in Health and Medicine who recommend a societal perspective and a 3% discount rate [65]. Parameter uncertainty analyses were undertaken for both the CBA and CUA by running 2000 iterations of the model using Monte-Carlo simulation using the Ersatz Excel add-in software (pigear, Brisbane, Australia https://www.epigear.com/index.htm) [66] (see Supplementary Materials, Table S1 for details of the distributions for model inputs). All results are reported as mean values with 95% uncertainty intervals (UI).

## 3. Results

**Figure 2:**
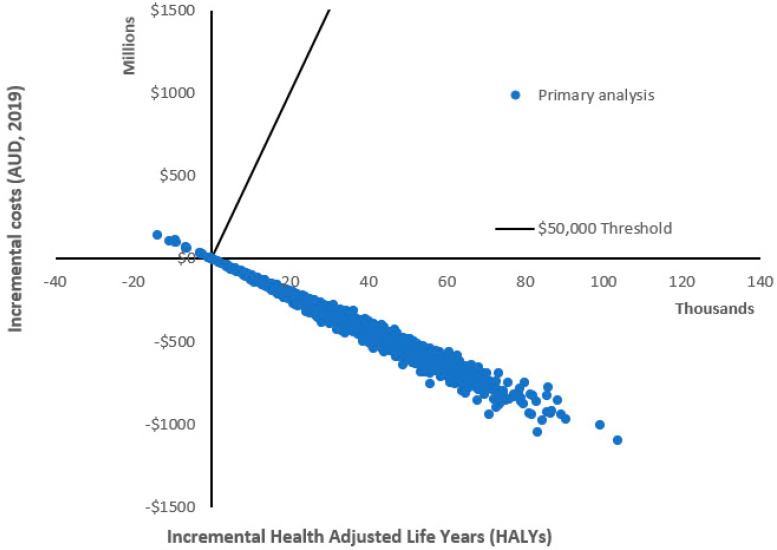
Cost-effectiveness plane for the primary cost–utility analysis.

The shelf tag intervention resulted in an estimated average change in energy intake per day of −115 kJ (95% UI: −234; −23) and in change in body weight of −1.09 kg (95% UI: −2.22; −0.21), which corresponds to a change in population BMI of −0.41 kg/m^2^ (95% UI: −0.82; −0.08) (Table 3). In the CBA, the change in BMI led to an estimated 50,923 HALYs gained (95% UI: 11,499; 101,399) and associated healthcare cost-savings of AUD 542.5 million (M) (95% UI: 121.6 M; 1.1 billion (B)) over the lifetime of the modelled population. The corresponding benefits in the CUA were estimated to be 36,930 HALYs gained (95% UI: 7527; 70,817) and healthcare cost-savings of AUD 406.5 M (95% UI: 81.5 M; 787.4 M). These differences in the CUA and CBA are due to differences in the discount rates in the reference case analysis (estimates for different discount rates are reported in Supplementary Materials, Table S5.1). Figure 2 shows the cost-effectiveness plane for the primary CUA analysis, demonstrating that 99.2% of model runs were dominant (cost-saving and health promoting).

The difference in perspective for the CBA and CUA produced marked differences in the cost of intervention implementation. The CBA analyses showed that the cost of implementing the three-year shelf tag intervention was approximately AUD 29.8 M (95% UI: 18.5 M; 44.1 M) with 98% of these costs accrued by supermarkets (Table 3). The key cost driver of supermarket costs was monitoring the shelf tag intervention followed by printing and installation. From the healthcare system perspective of the CUA, the total intervention cost was AUD 0.7 M (0.4 M; 1.1 M). Table 4 demonstrates the costs and benefits accrued by various stakeholders of the shelf tag intervention. Estimated costs borne by each supermarket chain and each state and territory government are shown in Supplementary Materials, Table S6. The clear beneficiaries of the intervention are consumers, the federal government and private health insurers who do not bear any of the costs, but accrue substantial benefits. The investment made by state and territory governments in implementing the intervention would be repaid by substantial healthcare cost-savings accrued by states and territories. Although there are several potential benefits that accrue to the supermarket industry, these have not been monetised and, therefore, it is unclear whether a voluntary shelf tag intervention would represent value-for-money from the retailer perspective.

In the CBA, the health gains valued using the VSLY were estimated to be approximately AUD 17 B, and dwarfed the other monetised benefits, resulting in the NPV also being in the region of AUD 17 B and a BCR of 591. The NPV (AUD 3.2 B) and the BCR (114) remained positive in a sensitivity analysis assigning a lower value to health benefits (AUD 50,000 per HALY gained) (Supplementary Materials, Table S5.6). Both the CBA and CUA produced consistent decision recommendations showing that the shelf tag intervention compared to a ‘status quo’ comparator was dominant—demonstrating the potential for significant cost-savings and health gains over the lifetime of the modelled population.

In all scenarios tested in the sensitivity analyses, the intervention remained highly cost-effective (Supplementary Materials, Table S5). The sensitivity analyses showed that a shorter duration of intervention implementation and intervention effect (Supplementary Material, Table S5.2) was 55% less costly; however, it resulted in approximately 96% fewer HALYs gained, demonstrating that a large component of costs accrued in the first year, whilst benefits accrue over several years and, therefore, efforts to maintain the intervention would enhance cost-effectiveness. The mandatory intervention was the most costly scenario (AUD 201 M); however, it also produced the highest NPV (AUD 1.79 B) and BCR (917). The results of all sensitivity analyses are reported in Supplementary Materials, Table S5.

## 4. Discussion

This study found that a shelf tag intervention promoting healthier packaged food products is potentially highly cost-effective—resulting in health gains and cost-savings over the lifetime of the modelled population. To our knowledge, this is the first economic evaluation of a shelf tag intervention and adds to the limited existing literature of the value-for-money of healthy food retail interventions [70]. The shelf tag intervention aligns well with the Australian National Obesity Strategy 2022–2032 that aims to work in partnership with supermarket retailers to “Improve nutrition information to help consumers make healthier choices at the time of purchase” [71]. Given the potential for the shelf tag intervention to offer excellent value-for-money, it should be considered as one of the components of a comprehensive approach to obesity prevention.

It is important that economic evaluations meet the needs of the decision-makers who may use them to inform preventive health policies. Interventions that affect businesses and have inter-sectoral impacts are considered ‘significant’ government policies and require Cabinet approval. CBA is required to inform Australian state government Cabinet decision-making [72,73]; however, health sector evaluations rarely use CBA [27]. This study conducted economic evaluations using both CBA and CUA. The CUA from a healthcare sector perspective would be inadequate to inform Cabinet decision-making, as industry costs (98% of total intervention costs) would be an important consideration. However, the outcomes of the CBA and CUA were consistent, with both analyses demonstrating that the shelf tag intervention is likely to represent value-for-money from both societal and healthcare sector perspectives. This is consistent with the findings of a review of studies that undertook both a CUA and a CBA, finding that for the majority of studies (78%), both analyses resulted in consistent adoption decision recommendations [27].

This study demonstrated the benefits of adopting a broader perspective. CBA has a clear intent to consider a broad range of costs and benefits; however, in practice, as with all forms of economic evaluation, measurement issues limited the scope of benefits valuation [74]. The CBA demonstrated that incorporating benefits other than health, such as consumer surplus (estimated to be five times greater than intervention costs and 26% of the estimated healthcare cost-saving in the primary CBA), may be important to consider. Further studies investigating consumer surplus associated with other labelling/promotion interventions such as incorporating nutrition and environmental benefits of food could be considered. 

The disaggregation of impacts by stakeholder group as recommended by the CBA framework for preventive health interventions [45] highlighted that although the benefits of the intervention in terms of health improvements and healthcare cost-savings were likely to be accrued by individuals and governments, the costs of the intervention were predominantly borne by supermarket retailers. The CBA underscored the need to establish the benefits of the intervention to retailers. Although we have provided this information qualitatively as part of the CBA (Table 4), further research is warranted to quantify and monetise these impacts. The CUA results were reported using both a narrow healthcare sector perspective (Table 3) and a broader societal perspective (Supplementary Materials, Table S5.7), which allows the comparison of findings of the shelf tag intervention with other CUA of obesity prevention initiatives that have used various evaluation specifications. Compared to a recent Australian study of obesity prevention interventions that were evaluated using a CUA with a limited societal perspective and a 3% discount rate, this shelf tag intervention would rank 9th out of the 16 cost-effective interventions evaluated [47].

In addition to the value-for-money assessment, there are other impacts that are important to consider prior to policy adoption [47]. We have demonstrated the distribution of costs and benefits of the shelf tag intervention across various stakeholder groups; however, there is a lack of empirical evidence on the equity impacts. The shelf tag study was implemented in regional Victoria in areas of high relative disadvantage and, therefore, the effect of the intervention is likely applicable to communities with similar socio-economic status; however, it is unclear how the effect might vary across communities with higher or lower socio-economic status and in other geographic locations. Importantly, the HSR scheme was developed to be understandable across socio-economic, culturally and linguistically diverse and low literacy/numeracy groups and is likely to be better understood than traditional nutrition labelling systems [75]. The environmental impacts of the shelf tag intervention, where paper tags are required to be regularly printed and replaced, have not been considered in this analysis. Future analyses from a broad societal perspective should consider environmental impacts of interventions, including the potential environmental benefits associated with reductions in chronic illness [76].

The shelf tag intervention was modelled as a voluntary initiative supported by Australian state governments, scaled up, and implemented across Australia. Given the ambiguity around the likely uptake of this intervention, various implementation scenarios were tested, including implementation in just one supermarket chain and mandatory implementation across all major chains. Although all scenarios were cost-effective, as expected, the mandatory implementation scenario resulted in the greatest NPV and BCR. Given that the majority of the cost of the intervention is upfront, with benefits accrued over the lifetime of the population, encouraging ongoing implementation enhances the value-for-money of the intervention. The Australian supermarket sector oligopoly is likely to increase the feasibility of widespread implementation of the intervention. The three largest supermarket chains in Australia (Woolworths, Coles, and Aldi) were early adopters of the voluntary HSR front-of-pack labelling scheme on their private label products [17], indicating that voluntary uptake of the intervention is feasible, particularly if there is sector-wide agreement that the intervention is both acceptable and effective. There are examples of successful roll out of shelf tag labelling systems by large retailers in other jurisdictions, but these have typically been where nutrient profiling logos have been incorporated into price tags and have not been implemented by multiple retail chains simultaneously [11].

The success and sustainability of a voluntary initiative over a three-year period is reliant on the potential benefits to supermarkets offsetting their costs. A recent process evaluation of a multi-component healthy supermarket retail intervention (Eat Well @ IGA) found that the component most often reported by staff to “not work well” was shelf tags (nominated by 17% of staff) due to the need to periodically replace tags that had fallen off [63]. Staff time for healthy retail intervention implementation has been highlighted as a barrier to intervention sustainability [63]. Despite the cost, retailers may be motivated to implement health-promoting interventions such as shelf tags to gain a competitive advantage, as shoppers are increasingly demanding healthier retail environments [13,63] and internationally, investors have started to compel supermarkets to increase sales of healthier products [77,78]. The use of the government-endorsed HSR system is likely to increase the feasibility of scaling up the intervention sector-wide. Feasibility will be further enhanced with strong government support, for example, the provision of a freely accessible database that contains the nutrient profile and HSR of packaged products (currently in development [79]), could reduce the time and cost to retailers associated with matching products to the HSR scheme.

A recent systematic review of economic evaluations of retail interventions showed that there is limited evidence on the cost-effectiveness of these interventions, with only eight published studies [70]. Only three economic evaluations (two cost-effectiveness analyses, with no long-term health outcome and one CUA) assessed the economic credentials of healthy food retail interventions in the supermarket setting [80,81,82]. All interventions focused on increasing the consumption of fruits and vegetables and the CUA study found that the intervention (a flyer identifying fruits and vegetables on sale, store signage to highlight discounted fruits and vegetables and a 50c voucher for fruits and vegetables) was not cost-effective [70]. This economic evaluation adds to the literature by quantifying the potential for health promoting supermarket interventions to impact population diets and long-term health outcomes. This analysis demonstrated that even small changes in energy intake could have significant population health impacts given the number of people impacted by supermarket-based interventions. This finding is consistent with findings from an Australian obesity prevention priority-setting study that found that regulatory interventions impacting the food environment with small impacts across the population were the most cost-effective [47].

This is the first economic evaluation of a novel supermarket-based shelf tag intervention promoting healthier packaged products. A key strength of this analysis was the broad range of costs and impacts, quantitatively and qualitatively included in the analysis. The use of a model that has been applied extensively to evaluate the cost-effectiveness of several Australian obesity prevention interventions [46,47] enhanced the validity of the findings. An extensive range of implementation scenarios and sensitivity analyses were conducted to demonstrate that under all tested scenarios, the intervention remained highly cost-effective.

There are several limitations of this analysis that should be considered when interpreting the results. Firstly, this economic evaluation was based on a single, short-term study in seven supermarket stores, and although the results were consistent in all intervention stores, further studies over a longer period and with a larger sample of stores, are required to increase confidence that the intervention effects are replicable and could be sustained over a longer period. Secondly, the shelf tag study was undertaken in regional Victoria; transferability of findings to the whole Australian context is also unknown. Finally, the results are reliant on a set of assumptions. The change in energy density of purchased packaged foods was modelled to directly translate to a change in energy consumption. We assumed that the quantity (weight) of packaged foods purchased and all other energy intake from all other sources remained unchanged. However, the shelf tag study indicated that there were small, but significant changes in the purchasing of non-packaged food products (fruit and vegetable sales reduced by a small but significant amount in the intervention compared to control stores [19]). Assumptions around how sales data translate to consumption were required because overall change in consumption was not measured in the shelf tag study [19]. Studies that have collected self-reported consumption and sales data have found inconsistent findings where changes in one outcome did not match the changes in the other [80,83]. These inconsistencies make it challenging to translate sales data to consumption, in order to assess the effectiveness of retail interventions on diet-related health outcomes. These challenges have been identified as common issues when conducting economic evaluations of retail interventions and further research in this area is needed [70]. 

## 5. Conclusions

The importance of intervening in the supermarket setting has been recognised in the Australian National Obesity Strategy. This study added to the limited economic evidence of nutrition-related interventions in the supermarket setting by demonstrating that shelf tags to alert customers of healthier packaged products are potentially highly cost-effective from both societal and healthcare sector perspectives, and should be considered as one of the components of a comprehensive approach to obesity prevention. For the evaluation of this non-health sector intervention, the CBA provided valuable insights, demonstrating that the majority of the costs fell on supermarkets. Therefore, opportunities to reduce costs and demonstrate benefits to this key stakeholder group require further investigation. Further studies confirming the longer-term effectiveness of the shelf tag intervention, conducted under trial conditions and preferably using robust measures of population consumption, are required to confirm the cost-effectiveness of this intervention.

## Figures and Tables

**Table 1:** Specifications of the primary cost–benefit and cost–utility analyses.

	CBA Primary Analysis	CUA Primary Analysis
**Perspective and Referent Group**	Societal, Australian population	Healthcare system
**Comparator/Base Case**	Status quo—no shelf tag intervention	
**Options for Appraisal**	Shelf tag intervention—3-year intervention and duration of effect	
**Time Horizon**	Lifetime account of healthcare costs and health impacts	
**Discount Rate**	3% (recommended by CBA framework [45])	5% (recommended by PBAC [21])
**Costs Included**	Supermarket industry, government	Government—health sector
**Benefits Included**	HALYs valued using VSLY Healthcare cost-savings Consumer surplus (WTP for HSR) Benefits to supermarkets (assessed qualitatively)	HALYs gained Healthcare cost-savings
**Decision Rules**	NPV/BCR	ICER: AUD per HALY gained
Sensitivity Analyses *	Univariate and multivariate sensitivity analyses (see Table 2)	
**Distributional Impacts and Other Considerations**	Equity and distributional impacts and other considerations described (qualitatively)	
**Reporting**	Disaggregate costs and benefits across subgroups (retailers, individuals, government, and various healthcare payers (e.g., state and federal governments, individuals and private health insurers).	Overall findings

Notes: AUD: Australian dollar; BCR: benefit–cost ratio; CBA: cost–benefit analysis; CUA: cost–utility analysis; HALY: health-adjusted life years; HSR: Health Star Rating; ICER: incremental cost–effectiveness ratio; NPV: net present value; PBAC: Pharmaceutical Benefits Advisory Committee; VSLY: value of a statistical life year; WTP: willingness to pay; * probabilistic sensitivity analyses (PSA) incorporated into all analyses.

**Table 2:** Description of sensitivity analyses.

	Cost–Benefit Analysis	Cost–Utility Analysis
**SA 1: Varied Discount Rate**	0%, 5%, 7%, 10%	0%, 3.5%
**SA 2: Lower Duration of Intervention Implementation and Effect**	1 year of intervention duration, 8 weeks of intervention effect (duration of the initial study)	
**SA 3: Limited Uptake by Supermarket Chains**	Only 50% of IGA stores implement the intervention, no implementation in other supermarket chains	
**SA 4: Exclude Consumer Surplus**	Remove consumer surplus benefit	No change
**SA 5: Shorter Time Horizon**	30 years	No change
**SA 6: Varied Monetary Valuation of Health Gains**	HALY gained: AUD 50,000; AUD 92,114; AUD 329,981 Life years gained: AUD 317,230	No change
**SA 7: Specifications of the Second Panel on Cost-Effectiveness in Health and Medicine [65]**	No change	Societal perspective—include implementation costs accrued by retailers. 3% discount rate.
**SA 8: Mandatory Intervention**	Cost of passing legislation in each state/territory. Increased implementation of 80% in all major supermarket chain stores. Implementation and monitoring costs and intervention effect for 20 years. Consumer surplus benefit was assumed to last for 3 years.	

Notes: AUD: Australian dollars, 2019 values; HALY: health-adjusted life year; SA: sensitivity analysis.

**Table 3:** Cost-effectiveness results, mean (95% UI).

	Cost–Benefit Analysis	Cost–Utility Analysis
**Population Change in Body Weight (kg)**	−1.09 (−2.22; −0.21)	
**Population Change in BMI (kg/m^2^)**	−0.41 (−0.82; −0.08)	
**Total HALYs Gained**	50,923 (11,499; 101,399)	36,930 (7527; 70,817)
**Total Intervention Costs**	AUD 29.8 M (18.5 M; 44.1 M)	AUD 0.7 M (0.4 M; 1.1 M)
Government Costs	AUD 0.7 M (0.4 M; 1.1 M)	AUD 0.7 M (0.4 M; 1.1 M)
Supermarket Costs	AUD 29.1 M (17.8 M; 43.5 M)	N/A
**Total Monetary Benefits**	AUD 16.8 B (3.9 B; 33.6 B)	N/A
Total Healthcare Cost-Savings	AUD 542.5 M (121.6 M; 1.1 B)	AUD 406.5 M (81.5 M; 787.4 M)
Consumer Surplus (Information Benefits of HSR)	AUD 139.8 M (8.5 M; 670.4 M)	N/A
Value of health gains	AUD 16.2 B (3.6 B; 32.2 B)	N/A
Net Costs for CUA *	N/A	− AUD 405.6 M (−786.8 M; −80.6 M)
**Net Present Value (NPV)**	AUD 16.8 B (3.8 B; 33.6 B)	N/A
**Benefit–Cost Ratio (BCR)**	591 (118; 1278)	N/A
**Mean Incremental Cost–Effectiveness Ratio (ICER)**	N/A	Dominant (Dominant to Dominant)
**Probability Intervention has Positive NPV/is Cost-Effective** **^β^**	99.6%	99.2%

Notes: AUD: Australian dollars, 2019 values; B: billions; BCR: benefit–cost ratio; BMI: body mass index; CUA: cost–utility analysis; HALYs: health-adjusted life years gained; HSR: Health Star Rating; ICER: incremental cost–effectiveness ratio; m: meters; kg: kilograms; M: millions; N/A: not applicable; NPV: net present value; UI: uncertainty interval. *Negative net costs represent savings. Dominant means that the intervention produces cost-savings and health gains compared to the no intervention comparator. ^β^ ICER below AUD 50,000 per HALY gain.

**Table 4:** Cost-effectiveness results, mean (95% UI).

Stakeholder Group	Costs, Mean (95% UI)	Benefits
**Supermarket Industry**	Total: AUD 29.1 M (17.8 M; 43.5 M) Design and matching: AUD 5.4 M (2.6 M; 8.9 M) Printing and installation: AUD 10.3 M (5.0 M; 17.8 M) Monitoring: AUD 13.4 M (6.4 M; 23.9 M)	Customer perceptions: The majority of customers exposed to healthy food retail interventions in supermarkets and grocery stores reported positive reactions to the intervention [60]. In the shelf tag study, 58% of surveyed customers who noticed the shelf tags reported that the shelf tags influenced their purchases [19]. Commercial viability: 84% of healthy food retail strategies in supermarkets and grocery stores either had a neutral or positive impact on measures of sales, revenue and profitability [60]. Retailer perspectives: In the shelf tag study, staff were positive about the intervention, and noted there was little work for the retailer. Retailers reported that the intervention was perceived positively by supplier representatives [29]. However, there were also reports that the shelf tags fell off easily [63]. Other potential benefits: Productivity gains from improved health of workforce * [67] See Supplementary Materials, Table S7 for details.
**Australian Federal Government (Healthcare Sector)**	AUD 0	Healthcare costs-saving: AUD 231.7 M Improved health from reduced chronic illness is predicted to improve productivity of the workforce resulting in increased taxes and reductions in welfare payments [68].
**Australian State Governments (Healthcare Sector)**	AUD 0.7 M (0.4 M; 1.1 M)	Healthcare costs-saving: AUD 150.3 M
**Private Health Insurers**	AUD 0	Healthcare costs-saving: AUD 44.5 M
**Individuals/Households**	AUD 0	Healthcare costs-saving: AUD 79.8 M Health benefits: AUD 16.2 B Consumer surplus (information value of HSR): AUD 139.8 M

Notes: AUD: Australian dollars, 2019 values; HSR: Health Star Rating; M: billi ons; UI: uncertainty interval. * Accrued by all businesses. Healthcare cost-savings accrued by the various stakeholders was informed by proportion of total health expenditure by governments, private health insurers and individuals as reported by the Australian Institute of Health and Welfare [69]. Note that total healthcare cost-savings from Table 3 are greater than the sum of the healthcare cost-savings across the reported stakeholders because 6.7% of healthcare costs are accrued by other non-government sources (i.e., injury compensation insurers and other sources of private income).

## Data Availability

All data supporting this research are provided within the manuscript and Supplementary Materials.

## Supplementary Materials

The following supporting information can be downloaded at: https://www.mdpi.com/article/10.3390/nu14091919/s1, Figure S1: Shelf tags installed in intervention stores; Table S1: Key model inputs; Table S2: Consolidated Health Economic Evaluation Reporting Standards (CHEERS) checklist; Table S3: Equations used in the cost–benefit and cost–utility analyses; Table S4: Description of sensitivity analyses; Table S5: Results of sensitivity analyses; Table S6: Costs accrued by supermarket chains and states and territories; Table S7: Description of benefits to supermarkets of healthy food retail interventions. References [84,85,86,87,88] are cited in the supplementary materials.
