# Supplementary material for: Cost–Benefit and Cost–Utility Analyses to Demonstrate the Potential Value-for-Money of Supermarket Shelf Tags Promoting Healthier Packaged Products in Australia
Source: Nutrients. 2022 May 3;14(9):1919. doi: 10.3390/nu14091919 (PMC9103654; doi:10.3390/nu14091919)
Supplement: Supplementary file 1 [file nutrients-14-01919-s001.zip › nutrients-1690974-supplementary.pdf]

Figure S1: Shelf tags installed in intervention stores

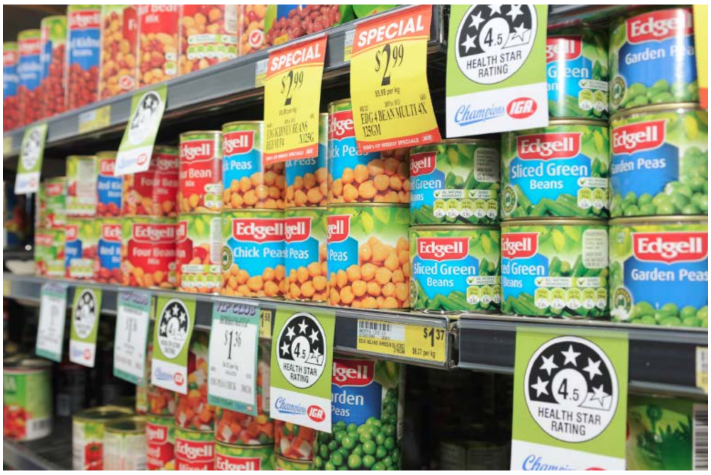

Table S1. Key model inputs.

**Modelling the intervention impact on population energy intake and weight**			
**Parameter**	**Value**	**Uncertainty distribution**	**Assumptions Notes/Source**
Proportion of all foods purchased at supermarkets	0.63	Pert (+/- 20% to inform the maximum and minimum)	Department of Agriculture, Fisheries and Forestry [1]
Packaged foods as a proportion of supermarket purchases	0.39	Normal (95% uncertainty interval 0.38, 0.4)	Australian shelf tag study baselines sales data
Market share of included supermarkets	0.84	Pert (+/- 20% to inform the maximum and minimum)	IBISWorld Pty Ltd [2]
Proportion of included supermarket stores that implement the intervention	0.50	Pert (+/- 50% to inform the maximum and minimum)	Assumption
Average daily energy consumption	Mean and SE by 5-year age and gender groups	Normal	Australian Health Survey 2011-12 [3]
**Modelling the cost of the intervention – cost to supermarkets**			
Shelf tags design	$2,051	Gamma	Shelf tag study estimates 1 week to design the tag. ABS Information media and telecommunications [4]*
Shelf tag printing cost per tag	$0.03	Pert (+/- 50% to inform the maximum and minimum)	Shelf tag study invoices inflated to 2019 values using the GDP Index from AIHW [5]
Frequency of tag replacement (each year)	26	Pert (+/- 50% to inform the maximum and minimum)	Assumed that the tags will need to be replaced every 2 weeks
Cost to access The George Institute for Global Health database to match nutrient profile to products	Not reported (this value is commercial in confidence)		Shelf tag study invoices inflated to 2019 values using the Total Health Price Index from AIHW [5]
Number of products in supermarkets	IGA: 10,958, SD 1,890	Gamma	Based on shelf tag study data for IGA stores
	Woolworths & Coles: 25,000	Pert ((+/- 20% to inform the maximum and minimum)	IBISWorld Pty Ltd [2]
Time to match products to nutrient profile database (per product, in hours)	0.017	Pert (+/- 50% to inform the maximum and minimum)	Based on researcher time in the shelf tag study.

Time for tag installation (per tag, in hours)	0.02 SD 0.01	Gamma	Based on researcher time in the shelf tag study.
Time to check/replace tag placement (per store, per week, in hours)	0.33	Pert (+/- 50% to inform the maximum and minimum)	Based on researcher time in the shelf tag study and adjusted to other c
Proportion of packaged foods with 4.5 or 5 stars	IGA & Aldi: 0.05	Gamma (95% UI: 0.03; 0.09)	Baseline data from intervention stores in shelf tag study.
	Woolworths & Coles: 14%	Pert (minimum informed by shelf tag study, +20% for maximum values)	Jones et al [6]
Additional products that require matching to nutrient profile database each year (as a proportion of total products)	0.32	Gamma	Based on shelf tag study
Cost to match product to nutrient profile database (per hour)	$49.74	Gamma	ABS Professional, Scientific and Technical Services [4]*
Costs to install and monitor tags (per hour)	$30.85	Gamma	ABS Retail trade [4]*
Number of stores per supermarket company	Woolworths: 995<br>Coles: 801<br>Aldi: 486<br>IGA: 1426	No variability included	2017 values reported in Euromonitor International [7]. No adjustments were made to reflect the number of stores in 2019. Although the number of stores generally increases over time, it could also decrease.
**Modelling the cost of the intervention – cost to state and territory governments**			
FTE required per year to support each supermarket head office (per state)	0.03		Calculated from "Fast Choices" evaluation [8].
FTE per store visit (year 1 only)	0.007		
Proportion of stores checked (in year 1 only)	7%	Pert (5% minimum, 50% maximum)	
Annual wage per FTE	$103,449	Gamma	ABS Professional, Scientific and Technical Services[b]*
**Additional inputs for cost-benefit analysis**			
VSLY	$317,230	Tested in one-way sensitivity analyses	Ananthapavan et al [9] inflated to 2019 values using the ABS Wage Price Index [10] as recommended by OBPR [11]
Consumer willingness to pay for HSR shelf tag (percentage of original price)	3.67%	Lognormal (95% UI: 3.34; 4.00)	Cooper et al [12]
Average annual spend on groceries per household	$6,974	Lognormal (95% UI: 6,577; 7,394	Birot et al [13] deflated to 2019 values using CPI for food and non-alcoholic beverages [14]
Number of households in Australia in 2019	9,654,900	Pert (+/- 20% to inform the maximum and minimum)	Extrapolated from ABS, Household and Family Projections [15].

*Labour on-costs (13%) [16] and 17.5% leave loading [17] included.
Notes: ABS: Australian Bureau of Statistics; AIHW: Australian Institute of Health and Welfare; A$: Australian dollars in 2019 values; CPI: consumer price index; FTE: Full time equivalent; GDP: gross domestic product; kJ: kilojoules; OBPR: Office of Best Practice Regulation; SD: standard deviation; SE: standard error; UI: uncertainty interval; VSLY: value of a statistical life year;

Table S2: Consolidated Health Economic Evaluation Reporting Standards (CHEERS) checklist

**Section/item**	**Item No**	**Recommendation**	**Section reported**
Title and abstract			
Title	1	Identify the study as an economic evaluation or use more specific terms such as "cost-effectiveness analysis", and describe the interventions compared.	Title
Abstract	2	Provide a structured summary of objectives	Abstract
Introduction			
Background and objectives	3	Provide an explicit statement of the broader context for the study.	Introduction: *paragraph 4*
		Present the study question and its relevance for health policy or practice decisions.	Introduction: *paragraph 4*
Methods			
Target population and subgroups	4	Describe characteristics of the base case population and subgroups analysed, including why they were chosen	Methods: *2.2. Modelling the intervention impact on population energy intake and weight*
Setting and location	5	State relevant aspects of the system(s) in which the decision(s) need(s) to be made.	Methods: *2.1. The intervention*
Study perspective	6	Describe the perspective of the study and relate this to the costs being evaluated.	Methods: *2.4 Overview of cost-effectiveness modelling; 2.4.1. Cost-benefit analysis modelling; 2.4.2. Cost-utility analysis modelling*
Comparators	7	Describe the interventions or strategies being compared and state why they were chosen.	Methods: *2.1. The intervention; 2.4 2.4 Overview of cost-effectiveness modelling*
Time horizon	8	State the time horizon(s) over which costs and consequences are being evaluated and say why appropriate.	Methods: : *2.4 Overview of cost-effectiveness modelling*
Discount rate	9	Report the choice of discount rate(s) used for costs and outcomes and say why appropriate.	Methods: *2.4.1. Cost-benefit analysis modelling; 2.4.2. Cost-utility analysis modelling; Table 1; Table 2.*
Choice of health outcomes	10	Describe what outcomes were used as the measure(s) of benefit in the evaluation and their relevance for the type of analysis performed.	Methods: *2.1. The intervention; 2.4 Overview of cost-effectiveness modelling*
Measurement of effectiveness	11a	Single study-based estimates: Describe fully the design features of the single effectiveness study and why the single study was a sufficient source of clinical effectiveness data.	Methods: *2.1. The intervention*
	11b	Synthesis-based estimates: Describe fully the methods used for identification of included studies and synthesis of clinical effectiveness data.	
Measurement and valuation of preference based outcomes	12	If applicable, describe the population and methods used to elicit preferences for outcomes.	Methods: *2.4 Overview of cost-effectiveness modelling*
Estimating resource use and costs	13a	Single study-based economic evaluation: Describe approaches used to estimate resource use associated with the alternative interventions. Describe primary or secondary research methods for valuing each resource item in terms of its unit cost. Describe any adjustments made to approximate to opportunity costs.	Methods: *2.3 Modelling the cost of the intervention; 2.3.1. Supermarket costs; 2.3.2 Government costs; Appendix Table A*
	13b	Model-based economic evaluation: Describe approaches and data sources used to estimate resource use associated with model health states. Describe primary or secondary research methods for valuing each resource item in terms of its unit cost.	Methods: *2.4 Overview of cost-effectiveness modelling*

		Describe any adjustments made to approximate to opportunity costs.	
Currency, price date and conversion	14	Report the dates of the estimated resource quantities and unit costs. Describe methods for adjusting estimated unit costs to the year of reported costs if necessary. Describe methods for converting costs into a common currency base and the exchange rate.	Methods: *2.3.2. Government costs; 2.4 Overview of cost-effectiveness modelling; Appendix Table A*
Choice of model	15	Describe and give reasons for the specific type of decision-analytical model used.<br>Providing a figure to show model structure is strongly recommended.	Methods: *2.4 Overview of cost-effectiveness*
Assumptions	16	Describe all structural or other assumptions underpinning the decision-analytical model.	Methods: *2.4 Overview of cost-effectiveness; references of where to find additional details is provided*
Analytical methods	17	Describe all analytical methods supporting the evaluation. This could include methods for dealing with skewed, missing, or censored data; extrapolation methods; methods for pooling data; approaches to validate or make adjustments<br>(such as half cycle corrections) to a model; and methods for handling population heterogeneity and uncertainty.	Methods: *2.4 Overview of cost-effectiveness; 2.4.3. Sensitivity analyses; Appendix Table A*
Results			
Study parameters	18	Report the values, ranges, references, and, if used, probability distributions for all parameters. Report reasons or sources for distributions used to represent uncertainty where appropriate. Providing a table to show the input values is strongly recommended.	*Appendix Table A*
Incremental costs and outcomes	19	For each intervention, report mean values for the main categories of estimated costs and outcomes of interest, as well as mean differences between the comparator groups. If applicable, report incremental cost-effectiveness ratios.	Results: *Table 3; Table 4*
Characterising uncertainty	20a	Single study-based economic evaluation: Describe the effects of sampling uncertainty for the estimated incremental cost and incremental effectiveness parameters, together with the impact of methodological assumptions (such as discount rate, study perspective).	*Appendix Table F*
	20b	Model-based economic evaluation: Describe the effects on the results of uncertainty for all input parameters, and uncertainty related to the structure of the model and assumptions.	*Appendix Table F*
Characterising heterogeneity	21	If applicable, report differences in costs, outcomes, or cost-effectiveness that can be explained by variations between subgroups of patients with different baseline characteristics or other observed variability in effects that are not reducible by more information.	N/A
Discussion			
Study findings, limitations, generalisability, and current knowledge	22	Summarise key study findings and describe how they support the conclusions reached. Discuss limitations and the generalisability of the findings and how the findings fit with current knowledge.	Discussion
Other			
Source of funding		Describe how the study was funded and the role of the funder in the identification, design, conduct, and reporting of the analysis. Describe other nonmonetary sources of support.	Financial support disclosure provided to journal

Conflicts of interest		Describe any potential for conflict of interest of study contributors in accordance with journal policy. In the absence of a journal policy, we recommend authors comply with International Committee of Medical Journal Editors recommendations.	The authors have declared that no competing interests exist.

Table S3: Equations used in the cost-benefit and cost-utility analyses

$$\boldsymbol{NPV} = \sum_{t=0}^{T} \left(\frac{\Delta B_t - \Delta C_t}{(1+r)^t}\right)$$

$$\boldsymbol{BCR} = \sum_{t=0}^{T} \left(\frac{\Delta B_t}{(1+r)^t}\right) \Big/ \left(\frac{\Delta C_t}{(1+r)^t}\right)$$

Where:

ΔB_t = Shelf tag intervention net monetary benefits in year t. All impacts of the intervention are included on the 'benefits' side of the equation)
ΔC_t = Shelf tag intervention net costs in year t (note in primary analysis, intervention duration is 3 years)
r = Real social discount rate (3% in primary analysis)
T = Number of years in the analysis period (lifetime of the modelled population)

$$\boldsymbol{ICER} = \sum_{t=0}^{T} \left(\frac{\Delta C_t}{(1+r)^t}\right) \Big/ \left(\frac{\Delta B_t}{(1+r)^t}\right)$$

Where

ΔB_t = Shelf tag intervention net benefits in year t, measured in HALYs
ΔC_t = Shelf tag intervention net costs in year t. All monetary impacts, including healthcare cost-savings are included in the calculation of the net costs (note in primary analysis, intervention duration is 3 years)
r = Real social discount rate (5% in primary analysis)
T = Number of years in the analysis period (lifetime of the modelled population)

Notes: BCR: benefit cost ratio; HALYs: health-adjusted life years; ICER: incremental cost-effectiveness ratio; NPV: net present value

Table S4: Description of sensitivity analyses

**SA 1: Varied discount rate**	Various discount rates reported in the CBA framework for preventive health interventions [18] and the PBAC guidelines [19] are tested.
**SA 2: Lower duration of intervention implementation and effect**	Given the shelf tag study was only implemented for 8 weeks, we tested a scenario where the effect of the intervention that is implemented over 1 year, has an effect on purchasing for 8 weeks.
**SA 3: Limited uptake by supermarket chains**	The primary analysis assumed 50% uptake of the shelf tag intervention across the targeted supermarket chains. In this scenario, it is assumed that only 50% of IGA stores implement the intervention – representing a low uptake scenario.
**SA 4: Exclude consumer surplus**	The consumer surplus estimated for the primary analysis is based on consumer WTP for the HSR on front-of-packs, and therefore may not directly reflect consumer WTP for shelf tags. This analysis tests the impact of removing consumer surplus from the cost-effectiveness results.
**SA 5: Shorter time horizon**	The CBA framework for preventive health interventions [18] recommends shorter time horizons in sensitivity analyses. A 30 year time horizon for costs and benefits is tested in this scenario.
**SA 6: Varied monetary valuation of health gains**	The CBA framework for preventive health interventions [18] recommends the testing of various estimates to value HALY gains: A$50,000; A$92,114; A$329,981. Given that the valuation of the VSLY relates to years of life lost, this was applied to the life years gained by the intervention rather than the HALY.
**SA 7: Specifications of the Second Panel on cost-effectiveness in Health and Medicine [20]**	The Second Panel on cost-effectiveness in Health and Medicine has recommended that one reference case includes a broad societal perspective. It is also recommended that a 3% discount rate. Other CUA of obesity prevention interventions have used these specifications [21].
**SA 8: Mandatory intervention**	The primary analysis assumed a voluntary intervention encouraged by state/territory governments. This scenario tests the impact of a mandatory policy. The additional cost to government included the cost of passing legislation in each state/territory [22] and the cost of monitoring compliance over 20 years. The cost to retailers was also extended for 20 years. However, the cost of accessing The George Institute for Global Health database was eliminated as the Food Standards Australia New Zealand agency is developing a database that will allow product matching and will be available from 2023 [23]. Informed by the compliance with mandatory legislation for school healthy food provision policies [24], 80% uptake and implementation was assumed. It was assumed that the consumer surplus lasted for 3 years.
Notes: A$: Australian dollars in 2019 values; CBA: cost-benefit analysis; CUA: cost-utility analysis; HALY: health-adjusted life years; HSR: health star rating; PBAC: Pharmaceuticals Benefits Advisory Committee; SA: sensitivity analysis; WTP: willingness to pay;	

Table S5: Results of sensitivity analyses

Table S5.1 SA1: Varied discount rate, mean (95% UI)

Cost-benefit analysis				
	**0%**	**5%**	**7%**	**10%**
**Population change in body weight (kg)**	-1.09 (-2.22; -0.21)			
**Population change in BMI (kg/m²)**	-0.41 (-0.82; -0.08)			
**Total HALYs gained**	92,825 (21,158; 183,302)	36,095 (6,081; 70,237)	27,216 (5,858; 54,283)	19,505 (4,664; 38,871)
**Total intervention costs**	$30.2M (18.7M; 44.7M)	$29.2M (18.2M; 43.8M)	$28.8M (18.2M; 41.5M)	$28.1M (17.5M; 41.7M)
Government costs	$0.7M (0.5M; 1.1M)	$0.7M (0.4M; 1.1M)	$0.7M (0.4M; 1.1M)	$0.6M (0.4M; 1.0M)
Supermarket costs	$29.6M (18.1M; 44.0M)	$28.6M (17.5M; 43.0M)	$28.1M (17.6M; 41.0M)	$27.5M (16.8M; 41.2M)
**Total monetary benefits**	$30.5B (7.0B; 60.3B)	$12.0B (2.1B; 23.3B)	$9.1B (2.1B; 18.0B)	$6.6B (1.6B; 13.0B)
Total healthcare cost-savings	$935.1M (211.1M; 1.9B)	$397.3M (67.9M; 772.6M)	$307.6M (69.6M; 612.2M)	$229.0M (54.2M; 438.0M)
Consumer surplus (information benefits of HSR)	$150.8M (9.2M; 752.3M)	$144.6M (9.2M; 742.6M)	$142.3M (8.6M; 728.1M)	$134.8M (8.4M; 649.3M)
Value of health gains	$29.4B (6.7B; 58.1B)	$11.5B (1.9B; 22.3B)	$8.6B (1.9B; 17.2B)	$6.2B (1.5B; 12.3B)
**NPV**	$30.5B (7.0B; 60.3B)	$12.0B (2.1B; 23.2B)	$9.1B (2.1B; 18.0B)	$6.5B (1.5B; 13.0B)
**BCR**	1,052 (243; 2,202)	428 (77; 913)	327 (74; 707)	243 (56; 517)
**Probability intervention has positive NPV**	99.6%	99.4%	99.6%	99.8%

Cost-utility analysis		
	**0%**	**3.5%**
**Population change in weight (kg)**	-1.09 (-2.22; -0.21)	
**Population change in BMI (kg/m²)**	-0.41 (-0.82; -0.08)	
**Total HALYs gained**	92,825 (21,158; 183,302)	46,595 (11,688; 93,929)
**Total intervention costs**	$0.7M (0.5M; 1.1M)	$0.7M (0.4M; 1.1M)
Government costs	$0.7M (0.5M; 1.1M)	$0.7M (0.4M; 1.1M)
Total healthcare cost-savings	-$934.4M (211.1M; 1.9B)	$500.3M (126.3M; 1.0B)
**Net costs for CUA***	-$934.4M (-0.9B; -176.6M)	-$499.6M (-1.0B; -125.8M)
**Mean ICER**	Dominant (Dominant to Dominant)	Dominant (Dominant to Dominant)
**Probability intervention is cost-effective$^{\beta}$**	99.3%	99.6%

BCR: benefit-cost ratio; BMI: body mass index; CUA: cost-utility analysis; HALYs: health-adjusted life years gained; HSR: Health Star Rating; ICER: incremental cost-effectiveness ratio; m: meters; kg: kilograms; M: millions; N/A: not applicable; NPV: net present value; UI: uncertainty interval; $: Australian dollars, 2019 values.
*Negative net costs represent savings. Dominant means that the intervention produces cost-savings and health gains compared to the no intervention comparator. $^{\beta}$ ICER below $50,000 per HALY gain.

Table S5.2 SA2: Lower duration of intervention implementation and effect, mean (95% UI)

	Cost-benefit analysis	Cost-utility analysis
**Population change in body weight (kg)**	-0.18 (-0.36; -0.04)	
**Population change in BMI (kg/m^2)**	-0.07 (-0.14; -0.01)	
**Total HALYs gained**	1,995 (395; 4,130)	1,454 (323; 2,868)
**Total intervention costs**	$13.5M (8.3M; 19.9M)	$0.5M (0.2M; 0.9M)
Government costs	$0.5M (0.2M; 0.9M)	
Supermarket costs	$13.1M (7.9M; 19.4M)	N/A
**Total monetary benefits**	$703M (163.0M; 1.4B)	N/A
Total healthcare cost-savings	$21.2M (4.1M; 43.1M)	$16.0M (3.7M; 31.6M)
Consumer surplus (information benefits of HSR)	$49.1M (3.2M; 240.3M)	N/A
Value of health gains	$632.8 (125.3M; 1.3B)	N/A
**Net costs for CUA***	N/A	-$15.5M (-31.3M; -3.2M)
**NPV**	$689.5M (150.2M; 1.4B)	N/A
**BCR**	54 (13; 120)	N/A
**Mean ICER**	N/A	Dominant (Dominant to Dominant)
**Probability intervention has positive NPV/is cost-effective$^{\beta}$**	99.4%	99.7%

BCR: benefit-cost ratio; BMI: body mass index; CUA: cost-utility analysis; HALYs: health-adjusted life years gained; HSR: Health Star Rating; ICER: incremental cost-effectiveness ratio; m: meters; kg: kilograms; M: millions; N/A: not applicable; NPV: net present value; UI: uncertainty interval; $: Australian dollars, 2019 values.
*Negative net costs represent savings. Dominant means that the intervention produces cost-savings and health gains compared to the no intervention comparator. $^{\beta}$ ICER below $50,000 per HALY gain.

Table S5.3 SA 3: Limited uptake by supermarket chains, mean (95% UI)

	Cost-benefit analysis	Cost-utility analysis
**Population change in body weight (kg)**	-0.10 (-0.19; -0.02)	
**Population change in BMI (kg/m^2)**	-0.04 (-0.07; -0.02)	
**Total HALYs gained**	4,554 (1,065; 9,144)	3,264 (612; 6,536)
**Total intervention costs**	$7.8M (4.3; 12.3)	$0.4M (0.4M; 0.6M)
Government costs	$0.4M (0.4M; 0.6M)	
Supermarket costs	$7.4M (3.9M; 11.9M)	N/A
**Total monetary benefits**	$1.5B (360.4M; 3.0B)	N/A
Total healthcare cost-savings	$48.7M (11.4M; 98.7M)	$36.0M (6.6M; 72.6M)
Consumer surplus (information benefits of HSR)	$12.7M (0.8M; 59.9M)	N/A
Value of health gains	$1.4B (337.9M; 2.9B)	N/A
**Net costs for CUA***	N/A	-$35.5M (-72.1M; -6.2M)
**NPV**	$1.5B (351.8; 3.0B)	N/A

**BCR**	200 (49; 407)	N/A
**Mean ICER**	N/A	Dominant (Dominant to Dominant)
**Probability intervention has positive NPV/is cost-effective[β]**	99.6%	99.5%
BCR: benefit-cost ratio; BMI: body mass index; CUA: cost-utility analysis; HALYs: health-adjusted life years gained; HSR: Health Star Rating; ICER: incremental cost-effectiveness ratio; m: meters; kg: kilograms; M: millions; N/A: not applicable; NPV: net present value; UI: uncertainty interval; $: Australian dollars, 2019 values. *Negative net costs represent savings. Dominant means that the intervention produces cost-savings and health gains compared to the no intervention comparator. [β] ICER below $50,000 per HALY gain.		

Table S5.4 SA 4: Exclude consumer surplus, mean (95% UI)

	**Cost-benefit analysis**
**Population change in body weight (kg)**	-1.09 (-2.22; -0.21)
**Population change in BMI (kg/m^2)**	-0.41 (-0.82; -0.08)
**Total HALYs gained**	50,923 (11,499; 101,399)
**Total intervention costs**	$29.8M (18.5M; 44.1M)
Government costs	$0.7M (0.4M; 1.1M)
Supermarket costs	$29.1M (17.8M; 43.5M)
**Total monetary benefits**	$16.5B (3.0B; 33.0B)
Total healthcare cost-savings	$542.5M (121.6M; 1.1B)
Consumer surplus (information benefits of HSR)	$0
Value of health gains	$16.2B (3.6B; 32.2B)
**NPV**	$16.5B (3.0B; 32.9B)
**BCR**	578 (108; 1,222)
**Probability intervention has positive NPV**	99.4%
BCR: benefit-cost ratio; BMI: body mass index; HALYs: health-adjusted life years gained; HSR: Health Star Rating; m: meters; kg: kilograms; M: millions; NPV: net present value; UI: uncertainty interval; $: Australian dollars, 2019 values.	

Table S5.5 SA 5: Shorter time horizon, mean (95% UI)

	**Cost-benefit analysis**
**Population change in body weight (kg)**	-1.09 (-2.22; -0.21)
**Population change in BMI (kg/m^2)**	-0.41 (-0.82; -0.08)
**Total HALYs gained**	40,472 (8,094; 79,517)
**Total intervention costs**	$29.8M (18.5M; 44.1M)
Government costs	$0.7M (0.4M; 1.1M)
Supermarket costs	$29.1M (17.8M; 43.5M)
**Total monetary benefits**	$13.4B (2.8B; 26.4B)

Total healthcare cost-savings	$444.5M (87.8M; 868.0M)
Consumer surplus (information benefits of HSR)	$139.8M (8.5M; 670.4M)
Value of health gains	$12.8B (2.6B; 25.2B)
**NPV**	$13.4B (2.8B; 26.4B)
**BCR**	472 (101; 974)
**Probability intervention has positive NPV**	99.7%
BCR: benefit-cost ratio; BMI: body mass index; HALYs: health-adjusted life years gained; HSR: Health Star Rating; m: meters; kg: kilograms; M: millions; NPV: net present value; UI: uncertainty interval; $: Australian dollars, 2019 values.	

Table S5.6 SA 6: Varied monetary valuation of health gains, mean (95% UI)

	HALY threshold: $50,000	Low VSLY: $92,114	High VSLY: $329,981	Valuation of LYs: $31
**Population change in body weight (kg)**	-1.09 (-2.22; -0.21)			
**Population change in BMI (kg/m²)**	-0.41 (-0.82; -0.08)			
**Total HALYs gained**	50,923 (11,499; 101,399)			LYs: 33,247 (7,946; 65,983)
**Total intervention costs**	$29.8M (18.5M; 44.1M)			
Government costs	$0.7M (0.4M; 1.1M)			
Supermarket costs	$29.1M (17.8M; 43.5M)			
**Total monetary benefits**	$3.2B (0.9B; 6.4B)	$5.4B (1.2B; 10.4B)	$17.5B (4.2B; 34.4B)	
Total healthcare cost-savings	$542.5M (121.6M; 1.1B)			
Consumer surplus (information benefits of HSR)	$139.8M (8.5M; 670.4M)			
Value of health gains	$2.5B (0.9; 7.5)	$4.7B (1.0B; 9.2B)	$16.8B (4.0B; 33.2B)	$11.2B (2.7B; 22.2B)
**NPV**	$3.2B (0.9B; 6.3B)	$5.3B (1.2B; 10.3B)	$17.5B (4.2B; 34.4B)	$11.2B (2.7B; 22.2B)
**BCR**	114 (29; 252)	187 (38; 397)	613 (137; 1,308)	395 (91; 813)
**Probability intervention has positive NPV**	99.9%	99.5%	99.7%	99.5%
BCR: benefit-cost ratio; BMI: body mass index; HALYs: health-adjusted life years gained; HSR: Health Star Rating; m: meters; kg: kilograms; M: millions; NPV: net present value; UI: uncertainty interval; $: Australian dollars, 2019 values.				

Table S5.7 SA 7: Specifications of the Second Panel on cost-effectiveness in Health and Medicine, mean (95% UI)

	Cost-utility analysis
**Population change in body weight (kg)**	-1.09 (-2.22; -0.21)
**Population change in BMI (kg/m^2)**	-0.41 (-0.82; -0.08)
**Total HALYs gained**	50,923 (11,499; 101,399)
**Total intervention costs**	$29.8M (18.5M; 44.1M)
Government costs	$0.7M (0.4M; 1.1M)
Supermarket costs	$29.1M (17.8M; 43.5M)
Total healthcare cost-savings	$542.5M (121.6M; 1.1B)
**Net costs for CUA***	-$514.0M (-1.1B; -78.7M)
**Mean ICER**	Dominant (Dominant to Dominant)
**Probability intervention is cost-effective$^{\beta}$**	99.6%

BMI: body mass index; CUA: cost-utility analysis; HALYs: health-adjusted life years gained; ICER: incremental cost-effectiveness ratio; m: meters; kg: kilograms; M: millions; UI: uncertainty interval; $: Australian dollars, 2019 values;
*Negative net costs represent savings. Dominant means that the intervention produces cost-savings and health gains compared to the no intervention comparator. $^{\beta}$ ICER below $50,000 per HALY gain.

Table S5.8 SA 8: Mandatory intervention, mean (95% UI)

	Cost-benefit analysis	Cost-utility analysis
**Population change in body weight (kg)**	-1.74 (-3.32; -0.37)	
**Population change in BMI (kg/m^2)**	-0.65 (-1.23; -0.14)	
**Total HALYs gained**	545,146 (103,779; 1,004,120)	352,557 (80,368; 658,469)
**Total intervention costs**	$200.9M (138.0M; 280.8M)	$16.4M (13.6M; 21.8M)
Government costs	$17.4M (14.1M; 23.7M)	$16.4M (13.6M; 21.8M)
Supermarket costs	$183.5M (121.4M; 261.5M)	N/A
**Total monetary benefits**	$178.7B (34.0B; 329.7B)	N/A
Total healthcare cost-savings	$5.6B (1.0B; 10.2B)	$3.7B (872.1M; 6.8B)
Consumer surplus (information benefits of HSR)	$224.2M (15.9M; 1.0B)	N/A
Value of health gains	$172.9B (34.0B; 329.7B)	N/A
**Net costs for CUA***	N/A	-$3.7B (-6.8B; -857.5M)
**NPV**	$178.5B (33.8B; 329.5B)	N/A
**BCR**	917 (173; 1,825)	N/A
**Mean ICER**	N/A	Dominant (Dominant to Dominant)
**Probability intervention has positive NPV/is cost-effective$^{\beta}$**	99.3%	99.5%

BCR: benefit-cost ratio; BMI: body mass index; CUA: cost-utility analysis; HALYs: health-adjusted life years gained; HSR: Health Star Rating; ICER: incremental cost-effectiveness ratio; m: meters; kg: kilograms; M: millions; N/A: not applicable; NPV: net present value; UI: uncertainty interval; $: Australian dollars, 2019 values
*Negative net costs represent savings. Dominant means that the intervention produces cost-savings and health gains compared to the no intervention comparator. $^{\beta}$ ICER below $50,000 per HALY gain.

Table S6: Costs accrued by supermarket chains and states and territories

Supermarket industry costs	
**Company**	**Total cost for 3 year intervention**
Woolworths	$12.0M (6.4M; 20.2M)
Coles	$9.1M (4.9M; 15.3M)
Metcash (IGA)	$7.3M (4.1M; 11.4M)
Aldi	$0.7M (0.5M; 1.0M)
**Total**	**$29.1M (17.8M; 43.5M)**
$: Australian dollars, 2019 values.	

State government costs	
**State/Territory**	**Total cost for 3 year intervention**
New South Wales	$150,662 (83,382; 276,880)
Victoria	$126,857 (73,785; 226,383)
Queensland	$109,004 (66,600; 188,436)
Western Australia	$76,447 (53,583; 118,945)
South Australia	$63,494 (48,170; 91,303)
Tasmania	$46,341 (41,006; 55,250)
Australian Capital Territory	$44,241 (40,062; 50,826)
Northern Territory	$42,140 (39,029; 46,313)
**Total**	**$658,182 ($447,137; 1.1M)**
$: Australian dollars, 2019 values.	

Table S7: Description of benefits to supermarkets of healthy food retail interventions

<table>
<tr><td colspan="2">There is evidence that retailers who have engaged in healthy food retail strategies value their contribution to the health of their customers and the community, however the impact of interventions on business outcomes may be equally or more important [25]. Business outcomes include measures of customer perceptions, commercial viability, and retailer perceptions [25]. The evaluation of the Australian shelf tag study and the process evaluation of a 12-month RCT that followed the shelf tag study (Eat Well @ IGA healthy supermarket RCT) provided insights on how the shelf tag intervention performs on these other outcomes of interest to supermarket retailers. Eat Well @ IGA was a multi-component intervention, one component was the shelf tag intervention. The other components included i) promotional signage including trolley and basket signs, posters and shelf signs; and ii) local area, in store and social media promotion of the Eat Well @ IGA initiative [26]. The shelf tag study included 304 cross-sectional customer exit surveys [27] and the process evaluation of the Eat Well @ IGA study included 754 customer surveys and interviews with store managers and others involved in implementing the changes [28].</td></tr>
<tr><td>Customer perceptions</td><td>Includes customer satisfaction with intervention strategy and store environment, convenience, increased customer demand for promoted products, and increased consumer welfare [25, 29, 30].
A review of previous studies have shown that healthy food retail strategies implemented in grocery stores, including supermarkets, produce favourable outcomes related to customer perceptions with 61% of measured customer perceptions being favourable and 85% of customer perceptions being either neutral or favourable [25]
34% of customers recalled the shelf tags in the shelf tag trial [27]. 58% of customers who noticed the shelf tags, reported the shelf tags influenced their purchases [27]
93% of customers of the Eat Well @ IGA initiative reported that the supermarket chain should continue to encourage health eating [26]</td></tr>
<tr><td>Commercial viability</td><td>Measures of commercial viability and profitability outcomes are diverse but important to retailers [25, 29, 30].
Healthy food retail strategies in grocery stores including supermarkets, either had a neutral or positive impact on measures of sales, revenue and profitability with 23% reporting positive impacts and 61% reporting neutral impacts [25]
Store managers believed that measures of the perception of customers on the overall brand was an important commercial viability outcome [25, 26]. However, in this analysis we have assumed that the shelf tag intervention was scaled up to be implemented across all major supermarket chains, diminishing the impact of the intervention in terms of competitive advantage. However, if shelf tags were expected by customers due to their prevalence in other supermarket stores, then the lack of implementation may result in competitive disadvantage.
7% of customers reported increasing the frequency of shopping at the Eat Well @ IGA intervention store because of the intervention [26]
Eat Well @ IGA intervention stores reported no overall effect on profits [26]</td></tr>
<tr><td>Retailer perceptions</td><td>Retailer perceptions include staff satisfaction, convenience of intervention implementation and retailer perceptions of the impact of the strategy on customer health outcomes and contributions to meeting the needs of the community [25, 30].
Previous healthy retail interventions in grocery stores including supermarkets, have shown that overall, there is a positive impacts on retailer perceptions, with 71% reporting positive impacts on these outcomes [25].
In the shelf tag study, interviewed retail staff were very positive about the intervention, and noted there was little work for the retailer. Retail representatives reported that intervention was perceived as positive by supplier representatives [28].
The strongest support for the Eat Well @ IGA initiative amongst store managers was due to the perceived positive impact of the intervention on community health outcomes [26]. This analysis has quantified and demonstrated the great potential of the shelf tags intervention on community health outcomes. However, it is unknown whether store managers across larger supermarket chains would value the benefits to the community in the same way.
The overall staff perception of the shelf tag component of the Eat Well @ IGA initiative was positive (median rating of 5 on a Likert scale of 1 (strongly dislike) to 7 (strongly like)) [26].
However, there were aspects of the shelf tag component of the Eat Well @ IGA study that did not work well noting that the tags fell off easily [26].
Most staff reported not receiving any customer feedback related to the Eat Well @ IGA, but when they did, it was neutral or positive [26].
93% of retail staff believed that the Eat Well @ IGA initiative would be maintained</td></tr>
<tr><td>Productivity impacts</td><td>Improved health in the community resulting from reductions in obesity related chronic illness are likely to translate into improved health for the retail workforce who also live in the community
Studies have shown that workers with chronic illnesses have reduced productivity with increased absenteeism rates (6.34 times higher than people without chronic illness) and increased</td></tr>
</table>

	presenteeism (lower levels of productivity whilst at work) rates (2.36 times higher than those without chronic illness). Therefore the benefits of reducing chronic illness in the community is likely beneficial for all employers including supermarket retailers [31]

References

1. Department of Agriculture Fisheries and Forestry. FOODmap. An analysis of the Australian food supply chain. Canberra: Department of Agriculture Fisheries and Forestry,, Department of Agriculture FaF; 2012 ISBN 978-1-921575-46-4 (online).
2. IBISWorld. IBISWorld Industry Report G4111 Supermarkets and Grocery Stores in Australia. 2018 February 2018. Report No.: G4111
3. Australian Bureau of Statistics. ABS 43640DO001_20112012 Australian Health Survey: Nutrition First Results – Foods and Nutrients, 2011–12 — Australia. In: Australian Bureau of Statistics, editor. Camberra, Australia: Australian Bureau of Statistics; 2014.
4. Australian Bureau of Statistics. 6302.0 Average Weekly Earnings, Australia, May 2019. TABLE 10H. Average Weekly Earnings, Industry, Australia (Dollars) - Original - Persons, Full Time Adult Total Earnings Canberra: ABS; 2022 [updated 24/2/2022; cited 2022 25/02/2022]. Available from: https://www.abs.gov.au/statistics/labour/earnings-and-work-hours/average-weekly-earnings-australia/latest-release.
5. Australian Institute of Health and Welfare. Health expenditure Australia 2018-19. Canberra, Australia: Australian Institute of Health and Welfare; 2020.
6. Jones A, Shahid M, Neal B. Uptake of Australia's Health Star Rating System. Nutrients. 2018;10(8):997. PubMed PMID: doi:10.3390/nu10080997.
7. Euromonitor International. Retailing in Australia. 2018.
8. NSW Food Authority. Review of Fast-food Labelling Requirements ("Fast Choices'') Canberra: NSW Government, 2012.
9. Ananthapavan J, Moodie M, Milat AJ, Carter R. Systematic Review to Update 'Value of a Statistical Life' Estimates for Australia. International journal of environmental research and public health. 2021;18(11):6168. PubMed PMID: doi:10.3390/ijerph18116168.
10. Australian Bureau of Statistics. Wage Price Index, Australia. Table 1. Total Hourly Rates of Pay Excluding Bonuses: Sector, Original, Seasonally Adjusted and Trend
In: Australian Bureau of Statistics, editor. Canberra, Australia: ABS; 2021.
11. Department of Prime Minister and Cabinet. Best Practice Regulation Guidance Note Value of statistical life. In: Department of Prime Minister and Cabinet, editor. Canberra: Commonwealth of Australia; 2021.
12. Cooper SL, Butcher LM, Scagnelli SD, Lo J, Ryan MM, Devine A, et al. Australian Consumers Are Willing to Pay for the Health Star Rating Front-of-Pack Nutrition Label. Nutrients. 2020;12(12):3876. PubMed PMID: doi:10.3390/nu12123876.
13. Birot M. What is the average grocery bill? : Canstar Blue; 2021 [cited 2021 28/10/2021]. Available from: https://www.canstarblue.com.au/groceries/average-grocery-bill/.
14. Australian Bureau of Statistics. TABLES 3 and 4. CPI: Groups, Weighted Average of Eight Capital Cities, Index Numbers and Percentage Changes. In: Australian Bureau of Statistics, editor. Canberra, Australia: ABS; 2022.
15. Australian Bureau of Statistics. Household and Family Projections, Australia. In: Australian Bureau of Statistics, editor. Canberra, Australia: ABS; 2019.
16. Australian Bureau of Statistics. Labour Costs, Australia. In: Australian Bureau of Statistics, editor. Canberra, Australia: ABS; 2017.
17. Fair Work Ombudsman. Payment for annual leave Canberra: Fair Work Ombudsman,; 2016. Available from: https://www.fairwork.gov.au/leave/annual-leave/payment-for-annual-leave#.
18. Ananthapavan J, Moodie M, Milat A, Veerman L, Whittaker E, Carter R. A cost–benefit analysis framework for preventive health interventions to aid decision-making in Australian

governments. Health Research Policy and Systems. 2021;19(1):147. doi: 10.1186/s12961-021-00796-w.
19. Department of Health. Guidelines for preparing a submission to the Pharmaceutical Benefits Advisory Committee. Version 5.0 In: Department of Health, editor. Canberra: Commonwealth of Australia; 2016.
20. Neumann PJ, Sanders GD, Russell LB, Siegel JE, Ganiats TG. Cost-effectiveness in health and medicine. Second edition. ed: Oxford University Press; 2017.
21. Ananthapavan J, Sacks G, Brown V, Moodie M, Nguyen P, Veerman L, et al. Priority-setting for obesity prevention—The Assessing Cost-Effectiveness of obesity prevention policies in Australia (ACE-Obesity Policy) study. PLOS ONE. 2020;15(6):e0234804. doi: 10.1371/journal.pone.0234804.
22. Lal A, Mantilla-Herrera AM, Veerman L, Backholer K, Sacks G, Moodie M, et al. Modelled health benefits of a sugar-sweetened beverage tax across different socioeconomic groups in Australia: A cost-effectiveness and equity analysis. PLOS Medicine. 2017;14(6):e1002326. doi: 10.1371/journal.pmed.1002326.
23. Food Standards Australia and New Zealand. Australian Branded Food Database Canberra: Food Standards Australia and New Zealand,; 2022 [cited 2022 25/03/2022]. Available from: https://www.foodstandards.gov.au/science/monitoringnutrients/Pages/Branded-food-database.aspx.
24. Chronic Disease Prevention Directorate. Healthy Food and Drink School Principal Survey Report 2019. Canberra: Western Australian Department of Health,, Western Australian Department of Health; 2021.
25. Blake MR, Backholer K, Lancsar E, Boelsen-Robinson T, Mah C, Brimblecombe J, et al. Investigating business outcomes of healthy food retail strategies: A systematic scoping review. Obesity Reviews. 2019;20(10):1384-99. doi: https://doi.org/10.1111/obr.12912.
26. Blake MR, Sacks G, Zorbas C, Marshall J, Orellana L, Brown AK, et al. The 'Eat Well @ IGA' healthy supermarket randomised controlled trial: process evaluation. The international journal of behavioral nutrition and physical activity. 2021;18(1):36. Epub 2021/03/14. doi: 10.1186/s12966-021-01104-z. PubMed PMID: 33712022; PubMed Central PMCID: PMCPMC7953771.
27. Cameron AJ BA, Orellana L, Marshall J, Crino M, Charlton E, Ngan W, Ananthapavan A, Lindsay J, Blake M, Sacks G,. Health Star Ratings on supermarket shelf tags to promote sales of healthy packaged foods store-wide. Nutrients. 2022; Submitted.
28. Blake MR SG, Marshall J, Brown AK, Cameron AJ. . A successful intervention research collaboration between a supermarket chain, the local government, a non-government organisation, and academic researchers: The Eat Well @ IGA healthy supermarket partnership. In: Jourdan D PL, editor. The Handbook of Health Promotion Research. Cham, Switzerland: Springer; 2022.
29. Gupta A, Alston L, Needham C, Robinson E, Marshall J, Boelsen-Robinson T, et al. Factors Influencing Implementation, Sustainability and Scalability of Healthy Food Retail Interventions: A Systematic Review of Reviews. Nutrients. 2022;14(2):294. PubMed PMID: doi:10.3390/nu14020294.
30. Houghtaling B, Serrano EL, Kraak VI, Harden SM, Davis GC, Misyak SA. A systematic review of factors that influence food store owner and manager decision making and ability or willingness to use choice architecture and marketing mix strategies to encourage healthy consumer purchases in the United States, 2005–2017. International Journal of Behavioral Nutrition and Physical Activity. 2019;16(1):5. doi: 10.1186/s12966-019-0767-8.
31. Fouad AM, Waheed A, Gamal A, Amer SA, Abdellah RF, Shebl FM. Effect of Chronic Diseases on Work Productivity: A Propensity Score Analysis. Journal of Occupational and Environmental Medicine. 2017;59(5):480-5. doi: 10.1097/jom.0000000000000981. PubMed PMID: 00043764-201705000-00009.
